# A Novel Program to Improve Cognitive Function in Individuals With Dementia Using Transcranial Alternating Current Stimulation (tACS) and Tutored Cognitive Exercises

**DOI:** 10.3389/fragi.2021.632545

**Published:** 2021-03-12

**Authors:** Zahra Moussavi, Kazushige Kimura, Lonnie Kehler, Cristina de Oliveira Francisco, Brian Lithgow

**Affiliations:** University of Manitoba, Biomedical Engineering, Winnipeg, MB, Canada

**Keywords:** cognitive exercises, mind triggers app, transcranial alternating current stimulation (tACS), dementia, tutoring, alzheimer's, gamma band stimulation

## Abstract

The effects of cognitive exercises on the healthy aging population is controversial. Transcranial alternating current stimulation (tACS) is considered a promising tool for modulating brain oscillation. Research is lacking on its long-lasting cognitive/therapeutic effect. This is the first pilot study to explore the effect of a regimen of cognitive exercises with and without tACS on older adults with dementia. The study groups were 28 individuals (age 56–83 years) enrolled into two groups: Exr Group, who received cognitive exercises only and the Exr + tACS Group who received tACS at 40 Hz simultaneously with cognitive exercises for a period of 4 consecutive weeks, 5 days/week, two 30 min-sessions/day; all the training sessions were tutored. The cognitive exercises were applied using the MindTriggers app. They were assessed at pre and post intervention and also one month after the end of trial (follow-up) with an independent assessment (WMS-IV) as the primary outcome measure. The results show significant cognitive improvement at post-intervention in both groups, while the Exr + tACS protocol lead to superior cognitive improvement at follow-up session. The most important outcomes of this study are: 1) The tutored repeated practice of the MindTriggers app exercises does significantly improve the cognitive functions of older adults with dementia and that that improvement lasts for at least one month after the end of the intervention, and 2) The application of tACS increases the positive effects of cognitive exercises with the positive effect lasting an even longer period of time than exercises alone; in other words we speculate that it may lead to long-term potentiation.

## Introduction

Due to advances in healthcare and improvements in life style, nowadays, people live longer. However, as longevity increases, cognitive abilities such as executive function, memory, reasoning, and processing speed deteriorate (Smith et al., 2009). While it can be part of normal aging (Boudreaus, 2010), a decline in cognitive function or associative and spatial memories can be considered a precursor to dementia, in particular Alzheimer’s (Didic et al., 2011). Currently there is no cure or "vaccine" for dementia, however, there is hope to delay the onset of the disease and/or slow its progression by keeping a brain wise-healthy life style. This hope is based on the neuroplasticity of the brain achievable even in old age as well as the individuals’ cognitive reserve (Stern, 2012). Constructing a “reserve capacity” has been suggested to help seniors maintain cognitive function (Baltes and Baltes, 1990). This theory was later supported by neuroimaging studies that showed increased contralateral hemispheric activity in right frontal regions for both working memory (Reuter-Lorenz et al., 2000) and episodic memory (Cabeza et al., 2002).

In recent years, there have been many programs and studies using different types of brain exercises claiming that they can enhance the cognitive function of older adults if they are used frequently and regularly (Willis et al., 2006; Garcia-Camupzano et al., 2013; Park and Bischof, 2013). However, improvements were observed mostly in the trained tasks (near-effect) with little or no transfer of cognitive functional enhancement to similar but untrained tasks (far-effect) (Karbach and Verhaeghen, 2014). Several review papers on meta-analysis of computerized training programs also found no evidence for far-effect for any working memory exercises on older adults (Karbach and Verhaeghen, 2014; Lampit et al., 2014). Another review study found no conclusive evidence of the effect cognitive exercises had on individuals with dementia and also questioned the amount of training required to receive any cognitive benefit from the exercises (Christie et al., 2017). A very recent study (Stojanoski et al., 2018), running a spatial training task and another similar testing task on 72 healthy participants (between 20 and 62 years), in particular, showed evidence against either a near-effect or far-effect gain from cognitive function training.

It is important to note that almost all studies on the effect of brain exercises on the cognitive function of adults and older adults had the participants doing the exercises on their own with no interaction with a trainer. In contrast to the above-mentioned study (Stojanoski et al., 2018), our previous and recent studies (Garcia-Camupzano et al., 2013; Garcia-Campuizano, 2013; Kehler et al., 2020) and also our recent study on the effect of brain exercises on older adults with memory problems (unpublished) have shown significant cognitive improvement in individuals with mild dementia when they received the brain exercises within a regimen comprising a tutored learning environment delivered on a daily basis; this improvement transferred to their daily life beyond the practiced exercises (the far-effect).

In addition to, or independent of brain exercises, some researchers have explored application of transcranial alternating current stimulation (tACS) on cognition. tACS is a relatively inexpensive, easy to administer and safe tool for non-invasive brain stimulation (Antal and Paulus, 2013); it utilizes the external application of oscillating electrical currents to influence cortical activity. The tACS-generated oscillating electric field has been demonstrated to both modulate and entrain the ongoing network oscillations in a frequency-specific manner (Antal and Paulus, 2013). The feasibility of using tACS for manipulating the phase, rhythm and power of neural oscillations via appropriate stimulation parameter choice (i.e., frequency, intensity, and anatomical location) has been demonstrated *in vitro* and *in vivo* experiments (Thut et al., 2017). The interest in tACS has increased significantly in the past decade. Its potential to influence neural oscillations in a frequency- and phase-specific manner offers the possibility to demonstrate causal relations between oscillations and behavior (Neuling et al., 2013; Bland and Sale, 2019). Furthermore, the largely untapped therapeutic potential of tACS has inspired a number of researchers to study its applicability as a treatment option for numerous neurological and psychological disorders (Kensinger et al., 2003). However, there has been little investigation into the potential effects of tACS on older adults (Antonenko et al., 2016) and no investigation on individuals with dementia. Moreover, while much of the research on tACS involves testing participants’ cognitive performance during or immediately following stimulation sessions (Jaušovec and Jaušovec, 2014; Meiron and Lavidor, 2014; Hoy et al., 2015; Antonenko et al., 2016), a cumulative (over a few weeks) tACS program has not been explored. In addition, the majority of the above-mentioned studies have applied tACS not only for a short duration but also without any other cognitive learning; only one study (Antonenko et al., 2016) applied tACS during a language learning paradigm. While, there is no study investigating the repeated training with tACS, from studies using transcranial direct current stimulation (tDCS), it is known that the stimulation alone without any cognitive learning does not lead to any long-lasting improvement (Huo et al., 2018).

In this pilot study, for the first time, we explore the effect of a regimen of cognitive exercises using the MindTriggers app (available on iPad AppStore) with and without tACS (called Exr + tACS and Exr groups, respectively) on older adults with dementia. The number of participants in our study is small but comparable with all other similar studies on the same topic. This is part of an on-going clinical trial (Moussavi, 2018) that repeats the training program every 4 months on individuals with mild to moderate dementia. This paper presents data and outcomes from the first round of tACS treatment program, whilst the repeated treatment data is being collected. We hypothesize that both treatment groups’ cognitive status will improve at post-intervention and that improvement will persist at least one month. In addition, we hypothesize that the tACS + Exr group will show more improvement than the Exr group. We investigated our hypotheses using the independent outcome measures at post-intervention and follow-up sessions compared to baseline.

## Method

### Participants’ Data

Data from 28 individuals with various degrees of dementia were adopted from the pool of data of an ongoing clinical trial studying the effects of cognitive exercises with or without tACS on the aging population (Moussavi, 2018). Participants of the above clinical trial were enrolled into tACS + Exr and Exr groups predominantly based on their tolerability to tACS and their preference; there was no randomization in group assignment. Participants who could not focus on exercises while receiving tACS or found tACS painful, were enrolled into Exr group. Therefore, our two groups of intervention are not matched in size or gender in this pilot study. However, as the clinical trial continues, we will generate balanced groups in the future.

All volunteers are assessed by Montreal Cognitive Assessment (MoCA) (Nasreddine et al., 2005). The MoCA is a 30-point test to assess cognitive function. This test takes approximately 20 min to administer in this population, and screens for mild cognitive impairment and dementia. A score of 26 or higher is considered as normal cognition. This test is used only for screening.

The study was been approved by the Biomedical Research Ethics Board of the University of Manitoba and all volunteers signed a consent form prior to enrollment into any of the study groups.

The selection of the data for this study was based on the following criteria:

- Age >60 years old
- A MOCA score of 7–25
- Be diagnosed with either mild cognitive impairment (MCI) or mild/moderate stage of a dementia subtype by a physician.
- Except for the dementia diagnosis, have no known diagnosis of major depression, bipolar disorder, schizophrenia, mood disorder, Parkinson’s disease, Huntington disease, ALS, MS, and/or any other neurological disorder.
- Have a Reading/writing/comprehension fluency in English
- Able to be enrolled in the 4-weeks, 5 days/week program of the clinical trial.
- Have no history of epileptic seizure or stroke.

Since the COVID19 pandemic, we have additionally offered this program Online but data of those participants have also been excluded from the analysis in this paper.

Data of 28 participants who met the above criteria, were selected for analysis in this study, out of which 3 felt the tACS as painful and 6 felt the tACS as tolerable but uncomfortable when doing the exercises at baseline session; thus, 19 were enrolled in tACS + Exr and 9 in Exr groups. Table 1 shows demographic information of the participants and their diagnosis, whose data were analyzed in this study.

**TABLE 1:** Participants Demographic information

Characteristics	tACS + Exr	Exr
N (M/F)	19 (13/6)	9 (7/2)
Age (years) (mean ± SE)	73.1 ± 1.8	69.5 ± 3
MoCA (mean ± SE)	18.4 ± 1.2	16.4 ± 1.2
Diagnosis	•8 AD (2 moderate, 6 mild)	•5 AD
	•3 LBD	•1 VD
	•1 FTD	•2 MCI
	•1 PCA	
	•1 VD	
	•4 MCI	
	•1 neurodegenerative dementia	

### Cognitive Exercises

We used the exercises within the MindTriggers app (Moussavi, 2020) delivered on an iPad. The exercises in this app were conceptually designed by the first author based on neuroplasticity of the brain and the most impaired cognitive functions in individuals with dementia, particularly Alzheimer’s. The app includes 7 different serious games with different difficulty levels. The focus of the games are on strengthening associative and spatial cognitive skills that deteriorate even in normal brain aging (Boundreaus, 2010). A significant decline in these two types of memory are symptoms of MCI, dementia and in particular Alzheimer’s. Associative memory is defined as the creation of new links between different items which were not related previously, and the later retrieval of these new associations (Buschkuehl and Jaeggi, 2010). Spatial memory includes the memory of geographical layout of an environment, and is important for orientation and navigation. Studies show recalling associative links between different items in older adults requires significant effort and energy (Buschkuehl and Jaeggi, 2010). These types of memory or cognition require a healthy connectivity between associated parts of the brain. For example, to recognize a familiar object and being able to name it; the right parietal lobe is activated to identify the object, it then communicates with the temporal lobe on the left side of the brain to name it. In this process, of course the hippocampus, which is central for long-term memory formation, and the prefrontal cortex which is involved in decision making, also become active and involved. Thus, an effective exercise for maintaining a healthy brain or improving the cognitive status of dementia population must target exercising these areas of the brain and their connectivity; that has been the premise of the designed games. Appendix A details the MindTriggers exercises and their scoring.

### Transcranial Alternating Current Stimulation (tACS)

We used the Soterix Medication tACS Stimulator (Model: 2001) on the tACS group. The stimulation was applied with a sinusoidal waveform at a frequency of 40Hz and a current amplitude of −0.75 mA to +0.75 mA (1.5 mA p-p). The active electrode was placed over the left dorsolateral prefrontal cortex (DLPFC) and the reference electrode was placed on the contralateral supraorbital area. Stimulation was applied simultaneously with the brain exercises per each session of treatment on participants in tACS + Exr group.

### Training/Treatment Protocol

The treatment protocol is to deliver the cognitive exercises of MindTriggers app (Moussavi, 2020) on an iPad with a trained tutor of our team on a daily basis for 4 weeks consecutively (excluding weekends, 20 sessions in total). Each daily treatment includes two 30 min duration cognitive exercises. The tACS + Exr group receive tACS simultaneously during the cognitive exercises. Each participant has a dedicated tutor throughout the period of the study.

### Assessments

The following assessments were run at baseline, post-intervention on first day of week 5 and a month after the end of the trial (follow-up). Although the cognitive exercises of MindTriggers have an analytic scoring system (Appendix A), we did not use those as metrics for measuring improvements for two main reasons: 1) the training sessions were tutored; thus, the scores do not reflect the true ability of the participants, and 2) the games’ scores, at best, measure the near-effect of the training program, which includes the practice effects of doing the same task repeatedly. We are interested in investigating the far-effect of the treatment program that can be assessed only by independent but relevant assessments as described below.

**Primary Outcome Measure: Wechsler Memory Scale (WMS IV) Older Adult Battery -** This assessment (Cullum and Larrabee, 2010) has six major memory indexes: Auditory Memory, Visual Memory, Visual Working Memory, Immediate Memory, Delayed Memory, and Recognition Memory. We calculated a total WMS score, which has a maximum score of 358, by summing all the sub-test scores, while we calculated memory index scores by summing related sub-test sections as shown in Table 2; note that memory indexes have overlap in terms of the sub-tests’ scores.

**TABLE 2:** WMS Memory index scores calculation.

Memory Index	Sub-test score components	Maximum score
Auditory Memory	Logical Memory I & II, and Verbal Paired Associates I & II	142
Visual Memory	Visual Reproduction I & II	86
Visual Working Memory	Symbol Span	50
Immediate Memory	Logical Memory I, Verbal Paired Associates I, and Visual Reproduction I	136
Delayed Memory	Logical Memory II, Verbal Paired Associates II, and Visual Reproduction II	92
Recognition Memory	Logical Memory II Recognition, Verbal Paired Associates II Recognition, Verbal Paired Associates II Word Recall, and Visual Reproduction II Recognition	80

**Secondary Outcome Measure: Egocentric Spatial Orientation -** This is a virtual reality (VR), focused on route finding and mapping in a cubic 3-story building with no landmark or cue; this test developed and evaluated in our lab for detection of spatial cognitive impairment (Byagowi et al., 2014; Ranjbar-Pooya et al., 2017). This test has two stages of assessment: 1) target localization from outside of the building, and 2) target finding inside the building. Most of those with MCI or dementia are not able to perform the target finding part as they find it very challenging. In this study, we only used the scores of the localization stage of the test using a laptop screen (not in an immersive mode).

**FIGURE 1:**
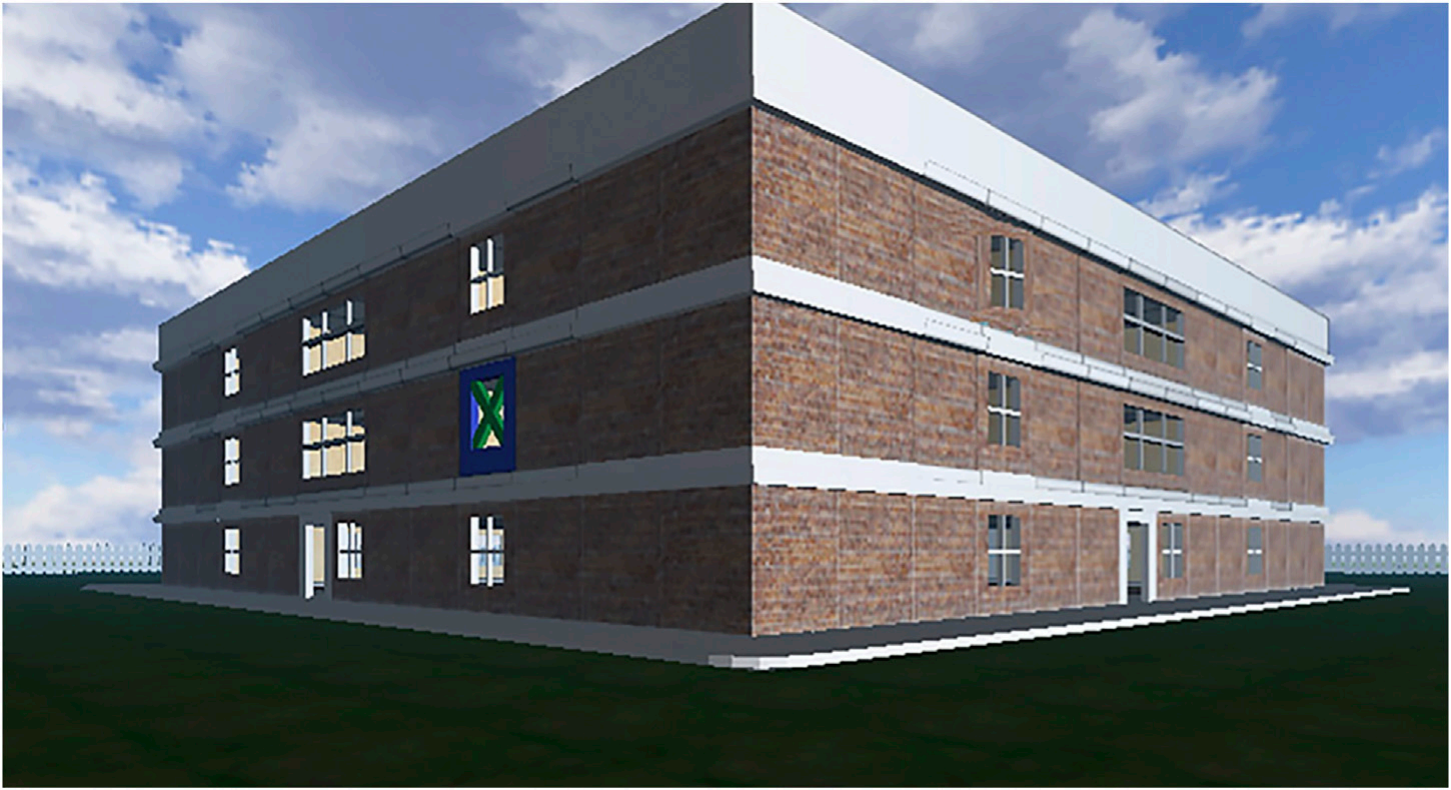
The VR building used for spatial orientation assessment.

In brief, the VR building in this test is a cubic symmetric landmark-less environment, with 4 rooms (with windows to outside) on each side of the building, 2 rooms/windows on each of the second and third floors in left and right corners of the wall (Figure 1). One of the windows in each trial of the eight trials is pseudo randomly marked with a big X mark as the target window (target appears on each front/back/left/right sides of the building in either left or right corners of either the second or third floor). The building is then rotated clockwise so that the participant can see every side of the building from outside. After a full rotation, the participant is asked to verbally say where the target window with the X mark is. This is called “target localization” stage, in which a target was placed on each left, right and back of the building twice in each corner of the wall. This stage of the test was run 6 times to cover the 6 different options as the target. If a participant identified the target correctly, s/he would receive 3 scores: 2 for identifying the target on a correct wall/side of the building and 1 for identifying the target on the correct corner of the wall. We do not assign a score for floor. As this test is run 6 times, the maximum correct score is 18.

**Montgomery Asberg Depression Rating Scale (MADRS) -** The MADRS (Montgomery and Asberg, 1979) is a ten-item rating scale for rating levels of depression. Each item is rated on a 7-point scale from 0 to 6, where 0 indicates absence of the symptom and 6 indicates extreme presence of the symptom. The time frame for the scale is the previous four weeks. MADRS was used to test for the presence of depression as a confounding variable.

### Statistical Analysis

We hypothesis that the daily training with MindTriggers’ cognitive exercises will improve the cognitive abilities of study participants in both tACS + Exr and Exr groups when assessed by the outcome measures pre to post-intervention. Moreover, we hypothesize the plausible positive effect of the cognitive exercises will last at least a month. Thus, we should not see a significant decline at Follow-up assessment with respect to immediate post-intervention. In addition, we hypothesize the group who receive tACS will be cognitively significantly better than the Exr group at follow-up assessment session.

Both groups’ data were checked for normality. If the outcome measures passed the normality, we used repeated measure Multivariate Analysis of Variance (MANOVA) and its post-hoc analysis and to investigate the above three hypotheses. If data did not pass the normality test, we used the equivalent non-parametric tests, the Wilcoxon Signed-Ranks test. In all statistical tests, *p = 0.05* was considered as significant.

## Results

Three participants in tACS group and one in non-tACS group did not have follow-up assessment scores due to the COVID19 pandemic but all had the post-intervention assessment.

**FIGURE 2:**
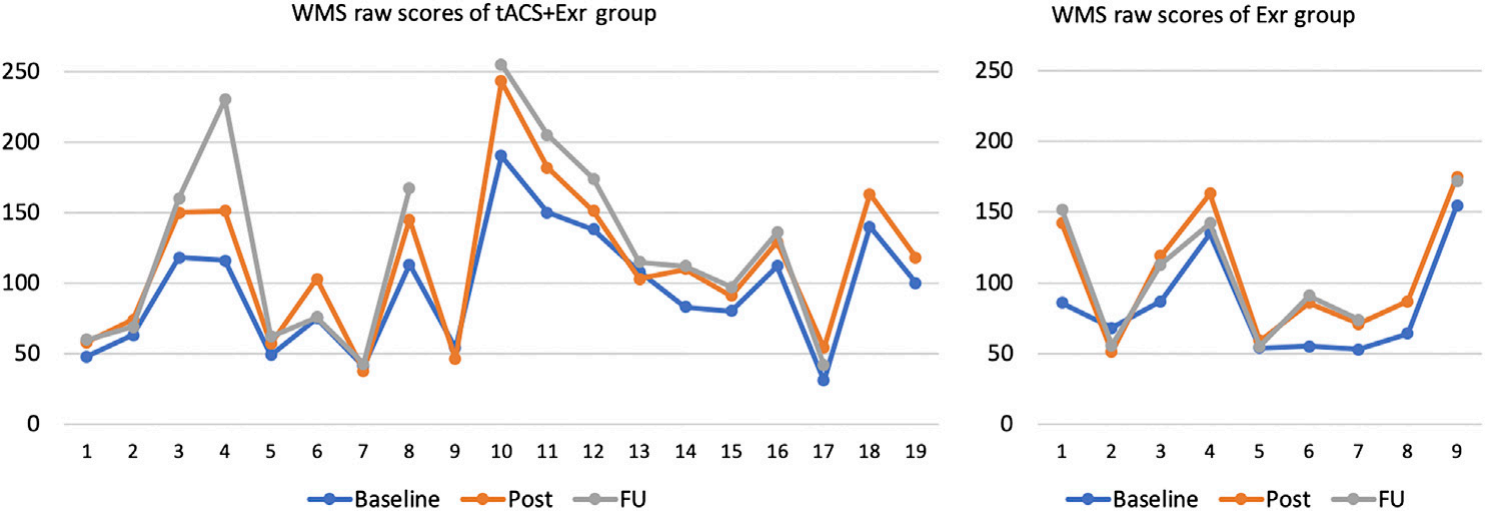
WMS raw scores of all participants of the two groups at baseline, post and follow-up sessions. Vertical axis is the WMS raw scores and horizontal axis is the participants’ number with no particular order. Note that three participants in tACS + Exr and 1 in Exr group missed the follow-up session assessment due to pandemic.

All participants of both groups except 3 in the tACS + Exr and 1 in the Exr groups, did improve in overall cognitive scores significantly when assessed by WMS-IV. Those 4 participants, showed negligible (only 1 or 2 scores of WMS-IV) decline in their scores. Figure 2 shows the WMS raw scores of all participants of the two groups at three assessment sessions. It is important to note that individuals with dementia decline gradually; thus, any program to help to avoid decline can be considered an improvement. The rate of decline, however, is neither linear nor the same among different dementia type. Therefore, it would be challenging to give an expected decline rate for our study participants as their cognitive impairment levels and causes varied widely.

None of the participants were suffering from depression at baseline and their depression scores remained approximately level throughout the sessions with small improvements at post-intervention. Table 3 shows the average and standard error of the WMS-IV total score, MADRS and Spatial scores at baseline, post-intervention and follow-up sessions.

**TABLE 3:** Participants’ performance data, Mean ± SE

Measure/Groups		Baseline	Post	Follow-up
**WMS-IV Total**	tACS	95.2 ± 9.7	114 ± 12.3	125.2 ± 16.9
	Non-tACS	75.3 ± 9.2	97.3 ± 13.4	101.5 ± 15.7
**MADRS**	tACS	3.7 ± 1.1	2.7 ± 1	1.5 ± 0.6
	Non-tACS	3.1 ± 0.7	1.9 ± 0.6	2 ± 1.1
**Spatial %**	tACS	49.3 ± 8.1	67.9 ± 6	64.7 ± 8.1
	Non-tACS	47.8 ± 15.5	66 ± 7.4	71.4 ± 13

**FIGURE 3:**
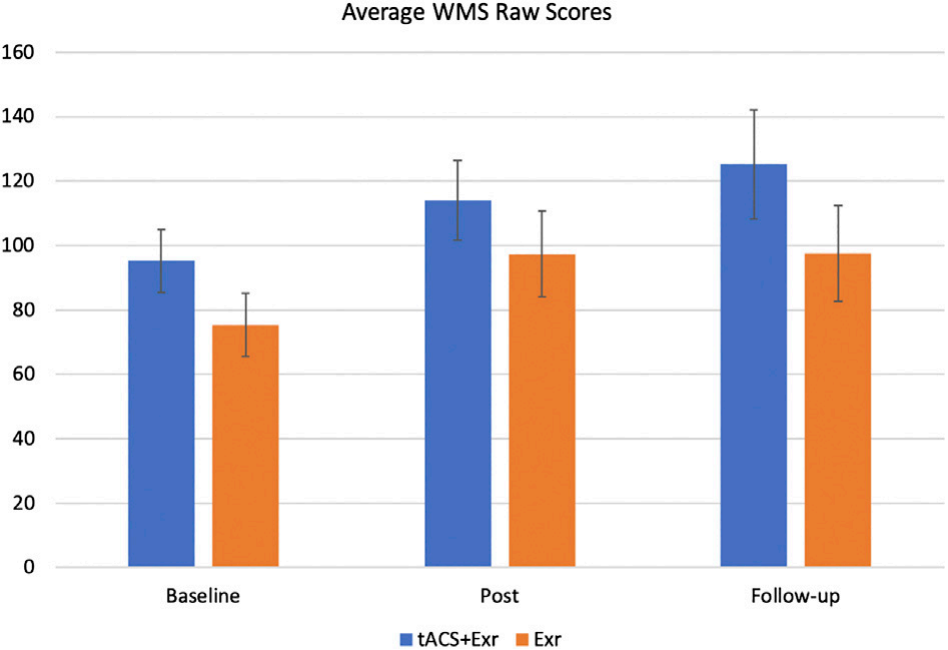
The average of primary outcome measure (WMS-IV) (mean ± SE) among participants of the two groups of tACS + Exr (*n* = 19) and Exr (*n* = 9) at baseline, post-intervention and follow-up. Both groups showed significant improvement at post-intervention respect to baseline; this improvement was still significant for tACS + Exr group but not for Exr group; see Table 4 for statistical analysis details.

**FIGURE 4:**
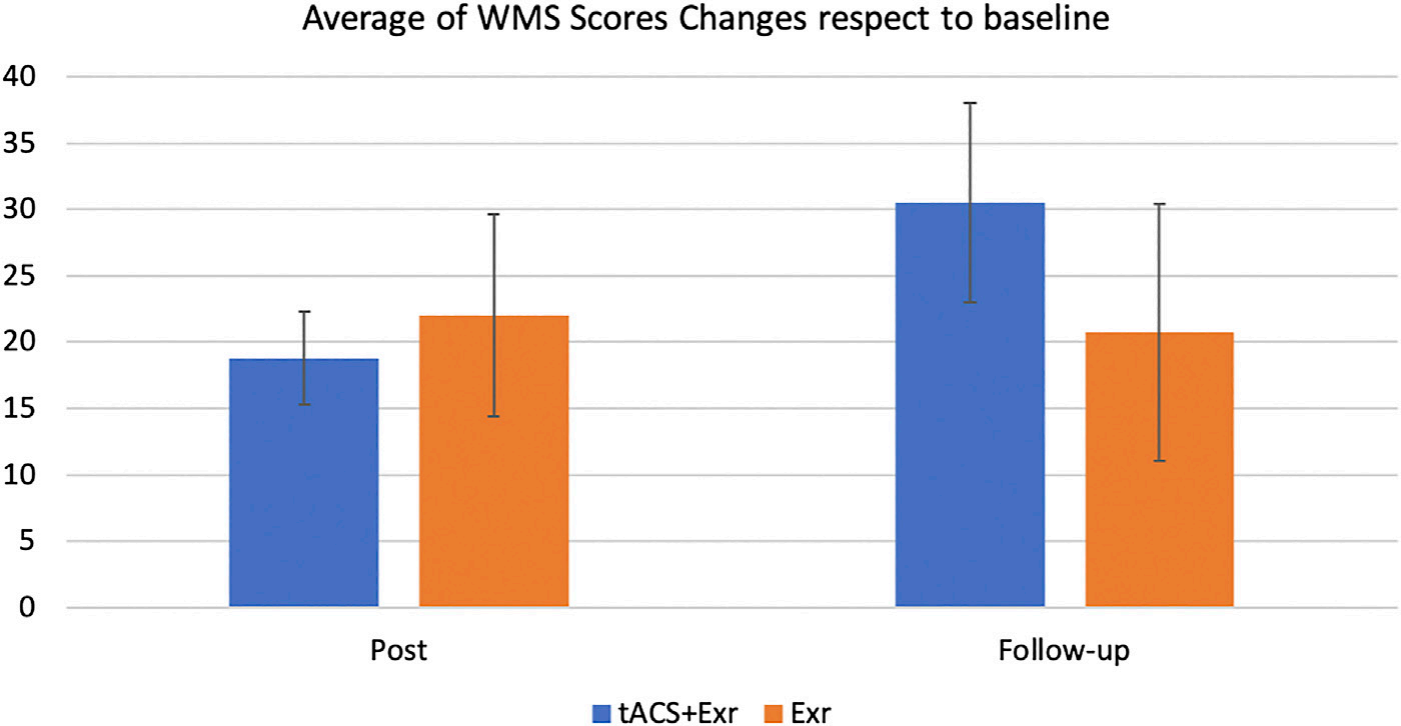
The average changes (mean ± SE) of the WMS-IV raw scores among participants of the two groups of tACS + Exr (*n* = 19) and Exr (*n* = 9) at post-intervention and follow-up respect to baseline. The changes between the two groups at follow-up session is evident but it was not statistically significant (*p* = 0.086); see Table 4 for details on statistical analysis details.

Repeated measure of MANOVA showed a significant improvement from baseline to post-intervention in both tACS + Exr and Exr groups (Figure 3), confirming the first hypothesis. As can be seen, both groups kept their improved state at the follow-up session, but interestingly the tACS + Exr group showed continued improvement confirming the second and third hypotheses. Figure 4 shows the changes (improvements) from baseline to post and follow-up sessions for each group, in which a significant difference between the two groups is evident at follow-up although it was not statistically significant. Details of statistical analysis are as follow.

We analysed the outcome measures data using multivariate analysis of repeated measures ANOVA with “Time” as within-subjects factor with 3 levels (baseline, post-intervention and follow-up) and “Group” as a Between-Subjects factors. Table 4 shows the significant effects and interactions. As for the *primary outcome measure (WMS-IV*), mixed ANOVA revealed a significant main effect of time (F (1.43, 30.05) = 13.997, *p* = 0.0002; effect size = 0.039). Thus, we performed post-hoc analysis with Bonferroni correction to investigate which group showed significant change in their WMS score at which time of the assessment (i.e., baseline, post-intervention and follow-up). The post-hoc analysis revealed that both groups showed significant improvements from baseline to post-intervention. At the follow-up assessment, the tACS + Exr group showed better retention of their improved status and even improved further at the follow-up session (a month after the end of intervention).

**TABLE 4:** Summary of the statistical tests in WMS scores. Adjusted *p*-values are bolded, and the *p*-value with *, ** and *** indicates significance at *p* < 0.05, *p* < 0.01 and *p* < 0.001, respectively. The bold values imply statistical significance.

Analysis	Statistic	*p*-value
Group * Time (interaction term)	F (1.43, 30.05) = 0.824	0.412
Group (tACS + Exr and Exr)	F (1, 21) = 0.068	0.797
Time (baseline, post-intervention and follow-up)	F (1.43, 30.05) = 13.997	0.0002***
tACS + Exr group from baseline to post-intervention	t (18) = −5.33	0.0001***
tACS + Exr group from baseline to follow-up	t (15) = −4.06	0.003**
tACS + Exr group from post-intervention to follow-up	t (15) = −1.83	**0.262**
Exr group from baseline to post-intervention	t (8) = −3.26	0.034*
Exr group from baseline to follow-up	t (6) = −2.07	**0.251**
Exr group from post-intervention to follow-up	t (6) = 0.519	**1.0**
Change from baseline to post-intervention between the two groups	t (12.66) = −0.39603	0.699
Change from baseline to follow-up between the two groups	t (13.404) = 0.84264	0.414
Change from post-intervention to follow-up between the two groups	t (20.87) = 1.8015	0.086

To investigate the last hypothesis, the changes in the WMS scores between the groups were compared using Welch two tailed *t*-test. The tACS + Exr group showed greater improvement than the Exr Group as expected although not statistically significant (*p* = 0.08). See Table 4 for details.

As one may claim the improvements might be due to decreased depression among participants, we also investigated the MADRS scores among the two groups. The Mixed ANOVA on MADRS scores did not reveal any significant interaction between the groups and time. Similarly, the change in the MADRS scores between the two groups did not differ significantly; see Table 5 for details.

**TABLE 5:** Summary of the statistical tests in MADRS scores.

	Statistic	*p*-value
Group * Time (interaction term)	F (2, 44) = 0.181	0.835
Group (tACS + Exr and Exr)	F (1, 22) = 0.079	0.781
Time (baseline, post-intervention and follow-up)	F (2, 44) = 2.206	0.122
Change from baseline to post-intervention between the two groups	t (21.775) = 0.054164	0.957
Change from baseline to follow-up between the two groups	t (14.794) = −0.53776	0.599
Change from post-intervention to follow-up between the two groups	t (14.61) = −0.055052	0.957

**FIGURE 5:**
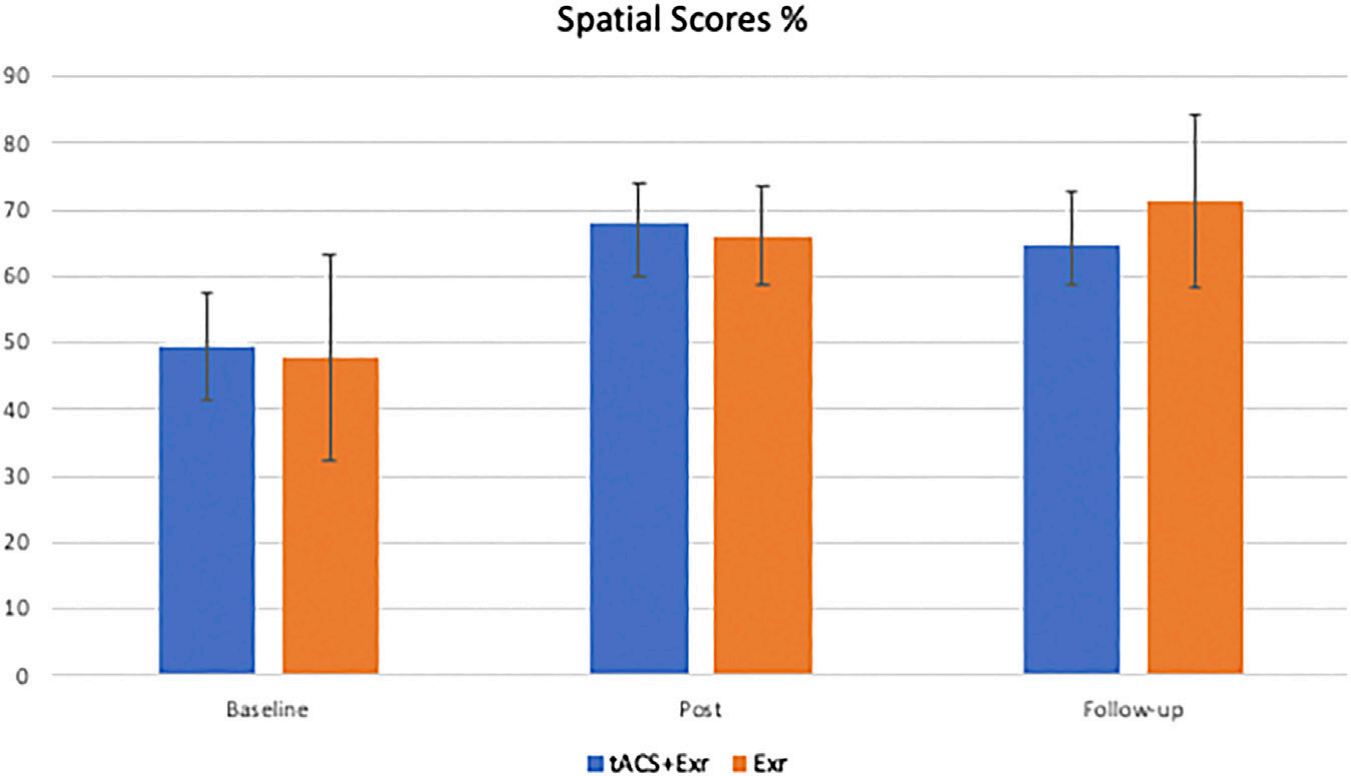
The average of secondary outcome measure (Spatial Orientation scores) (mean ± SE) among the two groups of tACS + Exr (*n* = 19) and Exr (*n* = 9) at baseline, post-intervention and follow-up.

The same analyses were applied for the secondary outcome measure, the *spatial assessment* scores. As Figure 5 shows, participants of both groups showed similar improvements on average after the intervention. However, both groups’ improvements at post-intervention and follow-up sessions were also the same. The Mixed ANOVA did not reveal a significant interaction between group and time (F (2, 38) = 0.249, *p* = 0.781; effect size = 0.004) and the main effect of the group (F (1, 19) = 0.107, *p* = 0.747; the effect size is 0.004), but revealed a significant main effect of time (F (2, 38) = 3.948, *p* = 0.028; effect size = 0.059). Similar to the analyses of WMS scores, a post-hoc analysis was designed with Bonferroni correction to investigate which group showed significant change in their spatial score at which time of assessment (i.e., baseline, post-intervention and follow-up). Despite the significant main effect on time, none of the pairwise comparisons revealed a significant change in either of the groups. Aside from the scores, the changes in the scores between the assessments were investigated. Similar to the analysis of WMS scores, the difference between the two groups were compared in terms of the changes in the spatial scores; however, unlike the primary outcome measure, no significant changes in spatial scores were found; see Table 6 for details.

**TABLE 6:** Summary of the statistical tests in spatial scores. Adjusted *p*-values are bolded, and the *p*-value with * means significance at *p* < 0.05. The bold values imply statistical significance.

	Statistic	*p*-value
Groups * Time (interaction term)	F (2, 38) = 0.249	0.781
Groups (tACS + Exr and Exr)	F (1, 19) = 0.107	0.747
Time (baseline, post-intervention and follow-up)	F (2, 38) = 3.948	0.028*
tACS + Exr Group from baseline to post-intervention	t (17) = −2.42	**0.082**
tACS + Exr Group from baseline to follow-up	t (13) = −1.23	**0.726**
tACS + Exr Group from post-intervention to follow-up	t (13) = 1.02	**0.978**
Exr Group from baseline to post-intervention	t (7) = −1.44	**0.582**
Exr Group from baseline to follow-up	t (6) = −1.53	**0.534**
Exr Group from post-intervention to follow-up	t (6) = −0.148	**1.0**
Change from baseline to post-intervention between the two groups	t (9.924) = −0.77787	0.455
Change from baseline to follow-up between the two groups	t (7.3245) = −1.5821	0.156
Change from post-intervention to follow-up between the two groups	t (10.214) = −1.5317	0.156

## Discussion

In this study, we used MindTriggers app (Moussavi, 2020) that includes a series of seven cognitive exercises (games) targeting strengthening the executive function of the brain, its left-right hemisphere connectivity and spatial orientation. We tested the efficacy of an intense cognitive exercises program (4 weeks, 5 days/week) on 28 individuals with dementia at mild and moderate stages in two groups: A) tACS + Exr Group: receiving tACS simultaneously with cognitive exercises, and B) Exr Group: receiving only cognitive exercises; both groups received the cognitive exercises with a trainer at our labs. The results overall showed significant improvement in the cognitive status of the participants that lasted at least for a month after the end of trial. Analysis of results at post-intervention respect to baseline confirms the first hypothesis that the cognitive exercises program has the main effect in improving the cognitive status of the participants as both groups improved similarly. On the other hand, those who did receive tACS simultaneously with cognitive exercises improved much more (*p = 0.043*) than those without tACS at follow up assessment. This interesting result imply the added positive effect of tACS when applied simultaneously with cognitive exercises in repeated sessions.

We applied tACS at 40 Hz in this study. Gamma band waves (>30 Hz) are known to be the fastest brain waves that are generated when the brain is learning a new concept and/or solving a problem. While research has not shown conclusively as to whether the dementia patients have a specific deficit in Gamma band activities (McDermott et al., 2018). it has been shown that stimulating the Gamma band activities can lead to an enhanced cognitive status in dementia patients (Silva et al., 2015), improved working memory (Hoy et al., 2015), increased endogenous attention (Hopfinger et al., 2017) and also may reduce intracerebral Tau protein burden (Dhaynaut et al., 2020); all these studies have had small sample sizes similar to our study. In this study, we observed that both groups (Exr and Exr + tACS) improved significantly and similarly post-intervention; however, the Exr + tACS continued to improve further during the month after the end of the intervention. The change between the two groups at follow-up did not reach the significance level, which might be due to small sample size. Nevertheless, the improving trend from post to follow-up in Exr + tACS group implies the application of tACS might have helped with long-term potentiation via enhanced modulation of neural circuitry to improve cognition.

On the other hand, application of tACS simultaneously with brain exercises does not seem to have the same effect on spatial cognition. As it is shown in Figure 4, both groups’ participants have improved their spatial cognition similarly at both post-intervention and follow-up sessions although neither changes were found statistically significant between the groups. This is an interesting outcome although due the limited sample size we have to be cautious in our interpretation. If it is verified in larger sample size, it might be due to the location of tACS application. We applied the tACS active electrode on the left DLPFC and its reference on contralateral supraorbital area; thus, mostly increasing the excitability of the prefrontal cortex. Spatial egocentric mapping, which was the type of spatial cognition exercised and tested in our study, involves several different regions of the brain but most importantly the posterior parietal cortex (Herweg and Kahana, 2018); that was not where we stimulated with tACS in this study. Moreover, one should consider that 6 out of the 7 games of MindTriggers app is focused on working memory including associative memory and only one game is dedicated to spatial memory. Likewise, the amount of time that a participant practiced the spatial memory was only 1/7 of the total time spent on cognitive exercises. It is possible that with more practice on that game, the results would become significant.

Aside from the more positive effect of tACS at follow-up, the results of this study is congruent with the outcomes of our previous studies (Garcia-Camupzano et al., 2013; Garcia-Campuizano, 2013) showing significant improvement in elderly with memory problems after going through tutored 20 sessions of brain exercises. It should be noted that all of our assessments have been independent of but relevant to the trained tasks; in fact, we measured the far-effect of our training protocol.

As mentioned in the Introduction, a recent study (Stojanoski et al., 2018) claims there is no general gain (no near or far-effect) of practicing brain exercises repeatedly in healthy individuals. Our studies’ results and in particular this study’s outcomes argues against that claim. It is worth comparing the similarities and differences of this study and that in (Stojanoski et al., 2018) although the participants of that study were healthy individuals (age 20–62) and our participants were older adults (age 56–83) with dementia. Given that if a program is effective for individuals with dementia it is also expected to be effective for healthy individuals. As there are no other similar studies to compare with our study, we compared the cognitive training programs in our study with that in (Stojanoski et al., 2018). For such comparison, we only compared the outcomes of EXR group of this study as the other study did not apply tACS.

The protocol of the study in (Stojanoski et al., 2018) was 1 month, 5 days/week (20 sessions) training although they accepted if a participant attended only 16 sessions of training. Similarly, they tested the participants pre- and post-interventions by independent assessment (but similar in concept) to investigate the far-effect of the training. However, aside the obvious differences such as age group (young adults vs. older adults in our study) and number of participants, there are several key differences between our study and that in ( Stojanoski et al., 2018) as follows.

The study in (Stojanoski et al., 2018)used only one game (we had seven different games) called “Token Search” which involves working and spatial memory. This game, although proven to be effective as for a measure of spatial and working memory, can become a boring game if played every day for a period of one month. Thus, it is not surprising that out of the 76 individuals recruited for the study in (Stojanoski et al., 2018) and who were paid upon completing the study, only 47 of them completed the study. In addition, the participants of the study in ( Stojanoski et al., 2018) were all young people with very little motivation other than being paid to participate in the study. On the other hand, among our participants, who were older adults with various degrees of dementia, there were a great desire to improve.

Most importantly, the participants in (Stojanoski et al., 2018) did the test by themselves as they were young healthy adults, while our participants all had a tutor/instructor sitting with them during the session and instructing them to choose a game and for how long, and help when necessary. This is a key factor behind the significant improvement of our participants post-treatment. It is in a way similar to hiring a personal trainer for exercising body and muscles, which normally results in better outcomes.

### Study Limitations

In this study, we did not have a matched size control group to measure the practice effect of the assessments. The main reason was the inability to recruit people in our target population willing to be only assessed and not receive the training. However, we had one MCI and one early-stage Alzheimer’s who were enrolled into the study but could not attend the daily sessions of the training due to living out of town; though, they did come in for the assessments. Those two participants’ data show no practice effect of the assessment; in fact, they showed a small decline in their scores at the subsequent assessments at 4 weeks and 8 weeks after baseline. In addition, in our previous study (Garcia-Camupzano et al., 2013), we had tested the practice effect of WMS-III in an age-and-gender-matched group using, which was found negligible among older adults who had some memory issues but were still considered to be cognitively healthy. Thus, in an MCI/dementia population of this study, the positive improvements observed most likely was not due to any practice effect of the outcome measures.

We also acknowledge the limited sample size of this study, similar to all previous studies in this subject area that use cognitive exercises and/or tACS. Recruitment of dementia patients for such study with a demanding protocol is challenging. Nevertheless, we hope in the near future we increase our sample size.

Aside from the limited sample size (similar to all other similar previous studies), another limitation of this study is that we did not have a sham (placebo) group for tACS. Also, the participants’ enrollment was not randomized between the two groups of the study. The main reason for lack of placebo group was that this study was funded by donations. For that reason, we felt morally responsible to give the participants the real treatment given the limited funds. We hope in the future, with a major funding, we can recruit enough participants to have a large randomized double-blind study.

## Conclusion

This paper, for the first time, presents the outcomes of our pilot study on repeated sessions of cognitive exercises with and without tACS in a regimen incorporating a tutored learning of older adults with various levels of dementia. The most important outcomes of this study are twofold: 1) The tutored repeated practice of the MindTriggers app exercises does significantly improve the cognitive functions of older adults with dementia and that improvement lasts for at least one month after the end of the intervention, and 2) The application of tACS improves the positive effects of cognitive exercises with the positive effect lasting a longer period of time; in other words we speculate that it may lead to long-term potentiation.

## Data Availability

The raw data supporting the conclusions of this article will be made available by the authors, without undue reservation.
